# Brain morphometric features predict depression symptom phenotypes in late-life depression using a deep learning model

**DOI:** 10.3389/fnins.2023.1209906

**Published:** 2023-07-19

**Authors:** Bing Cao, Erkun Yang, Lihong Wang, Zhanhao Mo, David C. Steffens, Han Zhang, Mingxia Liu, Guy G. Potter

**Affiliations:** ^1^College of Intelligence and Computing, Tianjin University, Tianjin, China; ^2^Department of Radiology and Biomedical Research Imaging Center, University of North Carolina at Chapel Hill, Chapel Hill, NC, United States; ^3^Department of Psychiatry, University of Connecticut School of Medicine, University of Connecticut, Farmington, CT, United States; ^4^Department of Radiology, China-Japan Union Hospital of Jilin University, Changchun, China; ^5^Department of Psychiatry and Behavioral Sciences, Duke University Medical Center, Durham, NC, United States

**Keywords:** cross-sectional late-life depression, deep learning, factor score prediction, cognitive impairment, Alzheimer's disease, structural MRI Frontiers

## Abstract

**Objectives:**

Our objective was to use deep learning models to identify underlying brain regions associated with depression symptom phenotypes in late-life depression (LLD).

**Participants:**

Diagnosed with LLD (*N* = 116) and enrolled in a prospective treatment study.

**Design:**

Cross-sectional.

**Measurements:**

Structural magnetic resonance imaging (sMRI) was used to predict five depression symptom phenotypes from the Hamilton and MADRS depression scales previously derived from factor analysis: (1) Anhedonia, (2) Suicidality, (3) Appetite, (4) Sleep Disturbance, and (5) Anxiety. Our deep learning model was deployed to predict each factor score via learning deep feature representations from 3D sMRI patches in 34 a *priori* regions-of-interests (ROIs). ROI-level prediction accuracy was used to identify the most discriminative brain regions associated with prediction of factor scores representing each of the five symptom phenotypes.

**Results:**

Factor-level results found significant predictive models for Anxiety and Suicidality factors. ROI-level results suggest the most LLD-associated discriminative regions in predicting all five symptom factors were located in the anterior cingulate and orbital frontal cortex.

**Conclusions:**

We validated the effectiveness of using deep learning approaches on sMRI for predicting depression symptom phenotypes in LLD. We were able to identify deep embedded local morphological differences in symptom phenotypes in the brains of those with LLD, which is promising for symptom-targeted treatment of LLD. Future research with machine learning models integrating multimodal imaging and clinical data can provide additional discriminative information.

## 1. Introduction

Major depressive disorder is the leading cause of disability worldwide (Ly et al., 2021), but also has distinct risks for older adults. Late-life depression (LLD), defined as depression occurring after age 60, is associated with higher rates of medical illness (Lyness et al., 2006), functional disability (Wassink-Vossen et al., 2019), and cognitive impairment (Panza et al., 2010) compared to older adults without depression. LLD is influenced by cumulative medical burden and age-related changes in brain structure and function, Jellinger (2022) which results in greater heterogeneity of symptom presentations and clinical outcomes (Hybels et al., 2011). A review of neuroimaging studies in LLD identified fronto-cingulate regions important to treatment outcomes of LLD (Zhukovsky et al., 2021); however, these studies do not characterize the types of depression symptoms that accompany these brain changes, which limits advances in linking brain structural changes to potential symptom-targeted treatments. Studies that combine depression symptoms and neuroimaging data can bridge this gap in knowledge by providing a more detailed characterization of the relationship between symptomatic and neural phenotypes in LLD.

Structural magnetic resonance imaging (sMRI) is a useful tool for examining clinical correlates and symptom phenotypes of LLD (Pimontel et al., 2021), particularly in combination with statistical methods that can optimize its predictive potential. While sMRI lacks some of the dynamic network-related information of functional MRI, it is more clinically accessible, has higher spatial resolution, and is less influenced by unknown factors in individual hemodynamic responses like medications and acute emotional state. Machine learning approaches have the potential use high-dimensional data from sMRI to identify novel associations between brain morphometric characteristics and symptom phenotypes (Uyulan et al., 2020; Zhang et al., 2022). Compared with latent variable models, which provide descriptive class information (Veltman et al., 2017), machine learning model can provide quantitative predictive information in high-dimensional MRI datasets. Machine learning approaches can reconcile issues around overlapping brain features in psychiatric conditions or symptom phenotypes (Zhuo et al., 2019) by extracting brain structural biomarkers that are accurate for diagnosis or classification (Gao et al., 2018). Whereas, many machine learning approaches like support vector machines (SVM), Gaussian process classifier (GPC), and linear discriminant analysis (LDA; Gao et al., 2018), rely on manually engineered (i.e., hand-crafted) neuroimaging features, deep learning approaches can extract information from raw data characteristics while demonstrating high classification accuracy and good generalization (Lin et al., 2018). Studies have used deep learning techniques with a variety of data sources to distinguish between depressed and non-depressed participants, but few studies have utilized sMRI data, and there is a need to apply deep-learning approaches to better understand the relationship between the brain structural and symptom heterogeneity of LLD.

The goal of the current study was to construct a deep learning model using sMRI morphometric features to predict depression symptom phenotypes in LLD. As symptom phenotypes, we used five symptom factors validated in LLD by previous research within our group (Potter et al., 2015). One challenge of applying deep learning methods to sMRI data is the large amount of data needed to learn a large number of parameters in deep neural networks during training (Litjens et al., 2017). In consideration of this challenge, we developed a novel “light” convolutional neuronal network model that focuses on 3D sMR image patches within 34 regions-of-interests (ROIs), and these ROIs are defined as a priori based on their correlation with LLD (Gunning et al., 2021) (Joseph et al., 2021). We predicted that we would be able to identify discriminative brain regions that would predict symptom phenotypes of LLD based on sMRI.

## 2. Method

### 2.1. Sample

Participants were enrolled in the Neurocognitive Outcomes of Depression in the Elderly Study (NCODE) (Steffens et al., 2004), which was approved by the Duke University Institutional Review Board. All participants in the parent NCODE study were age 60 or older at the time of enrollment. Depression diagnosis and screening of cognitive impairment was conducted by NCODE-trained psychiatrists using standardized assessment instruments and diagnostic algorithms, as described elsewhere (Steffens et al., 2004). Exclusion criteria included: (1) another major psychiatric illness including bipolar disorder, schizophrenia, or dementia; (2) alcohol or drug abuse or dependence; (3) primary neurologic illness, including dementia; and (4) contraindication to MRI scanning. Participants with comorbid anxiety disorders were not excluded, as long as the primary diagnosis was depression. For the current study, we included only participants with a time interval between clinical data and neuroimaging data < 6 months. This resulted in a sample size of 116 depressed participants (Table 1).

**Table 1:** Participant characteristics (sample size: *N* = 116).

Sex (% female)	68%
Race (% Caucasian)	77%
Age of depression onset (% >age 60)	16%
	**Mean (SD)**
Age	66.94 (6.32)
Education	14.61 (2.28)
MADRS	10.94 (9.27)
HDRS-17	15.44 (6.64)
Age of depression onset	36.99 (20.79)

MADRS, Montgomery-Asberg depression rating scale; HDRS-17, Hamilton depression rating scale-17 item.

### 2.2. Factor scores related to depression symptom phenotypes

As part of the enrollment assessment, an NCODE-trained geriatric psychiatrist interviewed participants with standardized clinical assessments, including the 17-item Hamilton Rating Scale for Depression Hamilton (HRSD17; Hamilton, 2000) and the Montgomery-Åsberg Depression Rating Scale (MADRS; Montgomery and Åsberg, 1979). These two widely used scales were combined for factor analysis in our previous work (Potter et al., 2015), the rationale of which was based on evidence that they capture a broader range of depression symptoms together than separately (Heo et al., 2007). The model in that study was a common factor analysis using principal component analysis (PCA) with varimax rotation to produce orthogonal factor scores. Factor interpretations and labels were listed as follows: Anhedonia and Sadness (Factor name: Anhedonia), Suicidality and Guilt (Factor name: Suicidality), Appetite and Weight loss (Factor name: Appetite), Sleep Disturbance, Anxiety and Tension (Factor name: Anxiety). All participants in the current study were included in that prior analysis, and their individual standardized factor scores from that analysis were applied to our current models.

### 2.3. MRI protocol and image preprocessing

Each participant completed a cranial MRI scan with an eight-channel parallel imaging head coil on a 3 Tesla whole-body MRI Siemens Trio system (Siemens Medical Systems, Malvern, PA). Proton density (PD), T1-weighted, T2-weighted, and fluid-attenuated inversion recovery (FLAIR) images were acquired. In the current study, we focused on T1-weighted sMRIs to predict the factor scores of depressed subjects. Since the intensity of the T1 displays a clear brain structure and it is useful for anatomy. Edema around the lesion areas is detectable using T2. T2-Flair is more sensitive to pathology than T2. Unlike T1 and T2 images, PD's signal intensity is related to tissue proton density. It has a high signal-to-noise ratio, and is used to visualize finely structured tissue. In the process of studying the causes of depression, we need to observe structural information in all ROIs rather than focusing on one lesion region, so the characteristics of the T1 modality are most suitable for the experiments in this paper. The T1-weighted image set was acquired using a 3D axial TURBOFLASH sequence with TR/TE = 22/7 ms, flip angle = 25°, a 100 Hz/pixel bandwidth, a 256 × 256 matrix, a 256 mm diameter field-of-view, 160 slices with a 1 mm slice thickness and Nex = 1 (no signal averaging), yielding an image with 1 mm cubic voxels in an 8 min, 18 s imaging time. All T1-weighted structural MR images were preprocessed via the following steps: (1) inhomogeneity correction with N4, (2) brain extraction, and (3) linear registration using FLIRT in FSL (with affine transformation only). Finally, all preprocessed MRI scans have the same size, containing 182 × 218 × 182 voxels with the image resolution of 1 × 1 × 1 mm^3^.

### 2.4. Regions of interest and patch extraction

**Figure 1:**
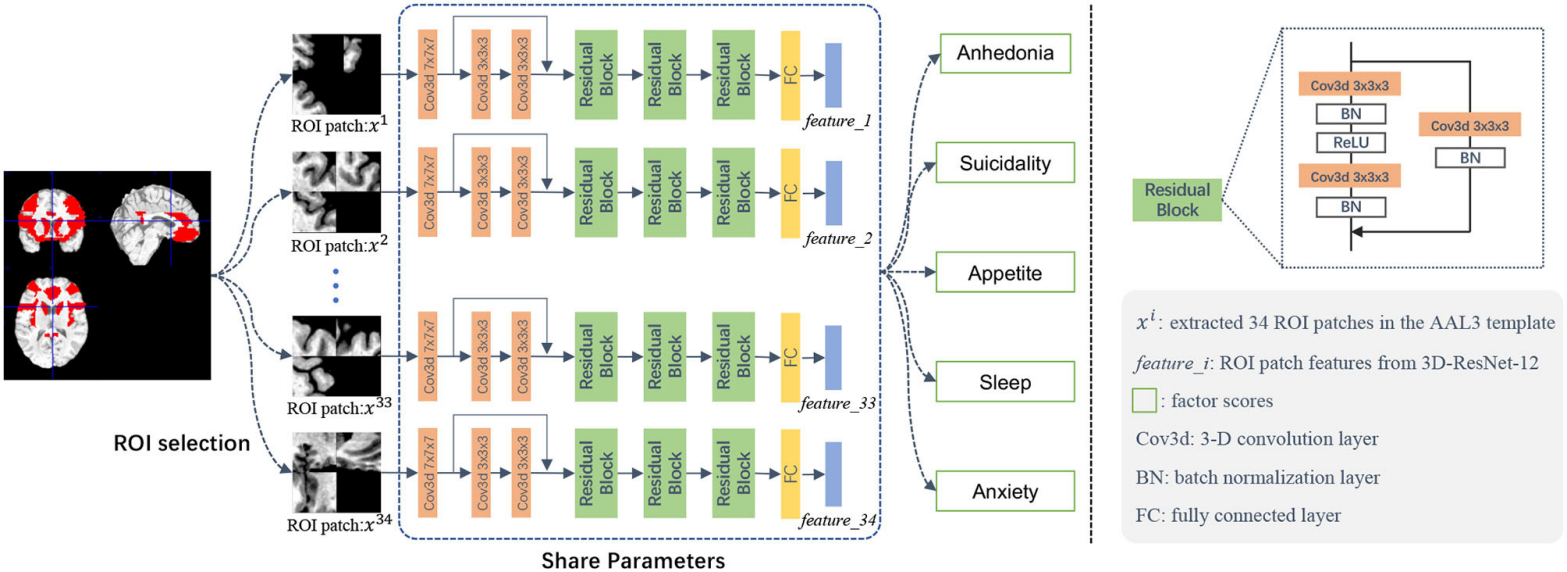
Illustration of the deep learning framework for structural MRI-based prediction of five late-life depression-associated symptom phenotype factor scores. This framework contains three major components: (1) ROI selection, (2) 3D image patch extraction, and (3) a deep neural network for prediction of five depression symptom phenotypes.

As illustrated in Figure 1, our modeling process has three main components: (1) ROI selection, (2) 3D image patch extraction, and (3) deep neural network prediction of depression symptom phenotype scores.

Based on available sample size, we developed a patch-based approach based on 3D MR image patches extracted from brain regions-of-interest (ROIs) that we selected a *priori* as commonly associated with LLD. As shown in Table 2, we used these 34 structural ROIs based on the AAL3 (Rolls et al., 2020) as masks to test whether we can predict our previously validated depression symptom phenotypes using structural neuroimaging data. These 34 ROI were organized into 10 testing sets (Table 2). To constrain model complexity and augment the data needs for deep learning models, we randomly extracted five different patches from each ROI. To extract the patch, we randomly selected patch centers within the ROI area. We randomized up to 20,000 times with the constraint of a preset distance between every two centers, which represents the overlap between two patches). Overlap increases with the size of ROI and varied from 50 to 75%; thus, our process ensured: (1) different patches were adequately separated from each other, but the centers of the patches were within each ROI, and (2) each patch could cover some voxels outside of the ROI to account for individual anatomical variability and potential registration error. We used a patch size of 32 × 32 × 32.

**Table 2:** Automated anatomical labeling (AAL) regions selected a *priori* for machine learning models.

**Anatomical description**	Label: aal2.nii.gz/ aal3.nii.gz*	**Abbreviation**
Anterior cingulate testing set		
Anterior cingulate cortex, subgenual	ACC_sub*	ACCsub
Anterior cingulate cortex, pregenual	ACC_pre*	ACCpre
Anterior cingulate cortex, supracallosal	ACC_sup*	ACCsup
Orbitofrontal PFC testing set		
Medial orbital gyrus	OFCmed	OFCmed
Anterior orbital gyrus	OFCant	OFCant
Posterior orbital gyrus	OFCpost	OFCpost
Lateral orbital gyrus	OFClat	OFClat
Dorsolateral PFC testing set		
Middle frontal gyrus	Frontal_Mid	MFG
Inferior frontal gyrus	Frontal_Inf_Oper	IFGoperc
Ventromedial PFC testing set		
Superior frontal gyrus, medial orbital	Frontal_med_orb	PFCventmed
Gyrus rectus	Rectus	REC
Posterior cingulate gyrus testing set	Cingulate_post	PCC
Hippocampus testing set	Hippocampus	HIP
Amygdala testing set	Amygdala	AMYG
Insula testing set	Insula	INS
Caudate nucleus testing set	Caudate	CAU
Nucleus accumbens testing set	N_Acc*	Nacc

PFC, prefrontal cortex. Each of the 17 regions includes (R) and left (L) hemispheres (*n* = 34). ^*^These labels indicate regions added to AAL3 (Rolls et al., 2020); otherwise labels reflect AAL2 convention (Rolls et al., 2015).

### 2.5. Deep learning model

The input of our deep learning model is sMRI image patches selected from the pre-defined ROIs (in the mask with values other than 0), and the output is each of the five depression factor scores for each subject. The estimation of each depression symptom factor score was trained separately as a single regression task. We used ResNet-18 (He et al., 2015) as the backbone of our model and developed a novel 3D CNN model, called 3D-ResNet-12, for our regression task. To accommodate a smaller sample size, we simplified the network architecture from 18 to 12 layers, as shown in the middle part of Figure 1. The 12 convolutional were followed by Batch Normalization (BN) layers (Ioffe and Szegedy, 2015), with the Rectified Linear Unit (ReLU) (Nair and Hinton, 2010) as the activation function following each BN layer. The skip connection can be expressed as a linear superposition and a nonlinear transformation of the input, and has shown effectiveness in addressing the vanishing gradient problem (He et al., 2015).

In the training phase, we fed the extracted ROI-based image patches to the regression network Φ. Note that we assign subject-level factor scores to all image patches in ROIs. For instance, for a specific subject, all patches extracted from the same ROI share the same Anxiety factor score. Denote *M* as the number of ROIs, *I* as the number of patches within each ROI, and the *i*-th patch as *x*^*i*^. In our experiments, we empirically set *M* to 34 and *I* to 5, respectively. The *patch-level* estimated score ${\hat{y}}_{patch}^{i}$ for the *i*-th patch can be computed as follows:


(1) $${\hat{y}}_{patch}^{i}=\Phi (x^{i}),i\in \{1,2,\cdots ,I\}$$

where Φ(·) denotes the forward computation process of the convolution network, and *i* denotes the patch ID.

The ROI-level estimated score ${\hat{y}}_{ROI}^{m}$ can be computed by the mean of all the patch-level estimated scores for the *m*-th (*m* = 1, ⋯ , *M*) ROI. The subject-level estimated score for the *n*-th subject ${\hat{y}}_{subjecr}^{n}$ can be acquired by averaging all the ROI-level estimated scores. Mathematically, the ROI-level and subject-level estimated scores are defined as follows:


(2) $$\begin{array}{l} {\hat{y}}_{patch}^{m}=\frac{1}{I}\overset{I}{\underset{i=1}{\sum }}{\hat{y}}_{patch}^{i} \end{array}$$

(3) $$\begin{array}{l} {\hat{y}}_{patch}^{n}=\frac{1}{M}\overset{M}{\underset{m=1}{\sum }}{\hat{y}}_{patch}^{m},m\in \left\{1,2,\cdots \;,M\right\} \end{array}$$

Our model maps the extracted patches to the estimated factor scores, and the regression loss L is based on the mean square error (MSE) and defined as follows:


(4) $$\begin{array}{l} L=||y_{patch}-{\hat{y}}_{patch}^{i}|{|}_{2}^{2} \end{array}$$

where *y*_*patch*_ denotes the real/ground-truth factor score vector (with each element denoting the real score for a specific training subject), ŷ_*patch*_ is a vector (with each element denoting the estimated/predicted score for a specific training subject), and $||\cdot |{|}_{2}^{2}$ denotes the *l*_2_ norm.

In the testing phase, we follow the same data preprocessing principle, and randomly extract 3D image patches from each ROI in each test MRI. The average of the estimated scores of the extracted patches from the same ROI from the ROI-based scores ŷ_*ROI*_. The average of all the ROI-level estimated scores form the subject-level estimated scores $\tilde{y}$ , defined as follows:


(5) $$\begin{array}{l} {\tilde{y}}_{ROI}^{m}=\frac{1}{I}\overset{I}{\underset{i=1}{\sum }}{\hat{y}}_{patch}^{i} \end{array}$$

(6) $$\begin{array}{l} \tilde{y}=\frac{1}{M}\overset{M}{\underset{m=1}{\sum }}{\hat{y}}_{ROI}^{m} \end{array}$$

where ${\tilde{y}}_{ROI}^{m}$ denotes the estimated scores of the *m*-th ROI, and $\tilde{y}$ denotes the final estimated scores of the test subject. The mean score of the extracted patches from the same ROI formed the estimated factor score at the ROI-level. We present the results at the ROI-level, which reflects the correlation between individual ROI and the factor score. This is expressed as Pearson correlation coefficient (*r*), with significance set at a *p* < 0.05.

### 2.6. Data partitioning

To ensure the generalization performance of the algorithm, we applied a 10-fold cross-validation (CV) strategy. This is supported by work showing that 10-fold CV provides a more stable performance between different data than leave-one-out cross-validation (LOOCV; Gao et al., 2018). Since each part is used as a test set and a training set, the over- and under-fitting problems can be well alleviated. In addition, it makes better use of the dataset because each data point is used for both testing and training, thus improving the performance of the model for testing and training, thus improving the generalization ability of the model. It can also help to select the best model parameters. By performing 10-fold cross-validation, the performance of the model with different parameters can be compared and the best combination of parameters can be selected.

To execute this, we (1) randomly split the dataset (*N* = 116) into 10-folds, (2) took each fold (10% of samples) as the testing set in turn, and (3) used the remaining 9-folds (90% of samples) in turn as the training set. The testing set was utilized to evaluate the performance of model generalization ability measurement.

### 2.7. Implementation

Five different 3D-ResNet-12 models were separately trained to predict factor scores, with each one corresponding to a specific factor score. For each model, we used patches from different ROIs to train the neural network. The mean square error (MSE) loss was used as the loss function. The batch size was set to 1,000, and the initial learning rate was set to 0.001. All models were implemented on PyTorch under the environment of Python 3.7 on the Ubuntu 18.04 system with NVIDIA TITAN Xp GPU.

The general regression process is implemented using least squares, while we implement a robust regression here based on the iteratively reweighted least squares (IRLS) to reduce the effect of outliers.

The general regression process can be described as the following equation, where x is the observed value, b is the expected value, *A* is the regression coefficient, and *e* is the error value defined as follows:


(7) $$\begin{array}{l} e=Ax-b \end{array}$$

And the optimization objective in the general regression is defined as:


(8) $$\begin{array}{l} ||e|{|}_{2}^{2}=\underset{i}{\sum }e_{i}^{2}=e^{T}e \end{array}$$

The IRLS then uses a weighted 2-norm to emphasize or de-emphasize certain components, where the diagonal array W is computed based on the last error e and therefore changes continuously.


(9) $$\begin{array}{l} ||e|{|}_{p}^{p}=\underset{i}{\sum }e_{i}^{\left(p-2\right)}e_{i}^{2}=\underset{i}{\sum }w_{i}^{2}e_{i}^{2}=||We|{|}_{2}^{2} \end{array}$$

## 3. Results

### 3.1. Depression symptom factors

Predictive modeling revealed specific networks associated with the five symptom phenotype factors. Regression errors were averaged for each of the 10 ROI sets across all 116 testing subjects. All the medians of MSE were smaller than 2, which demonstrates that the proposed model achieved good performance. The *p*-value is used to determine the risk level for rejecting the H0 hypothesis. In this paper, the H0 hypothesis is that the five factors are not related to LLD, so when the *p*-value is less than a certain threshold (generally 0.05), we can assume that the H0 hypothesis is not valid, that is, the five factors are significantly related to LLD. The *p*-value can be calculated by the formula of chi-square, where *O* is the observed value, *E* represents the expected value, and *i* is the number of samples, and then the chi-square distribution table can be queried according to the degrees of freedom in the hypothesis:


(10) $$\begin{array}{l} p=\overset{n}{\underset{i=1}{\sum }}\frac{{\left(O_{i}-E_{i}\right)}^{2}}{E_{i}} \end{array}$$

**Figure 2:**
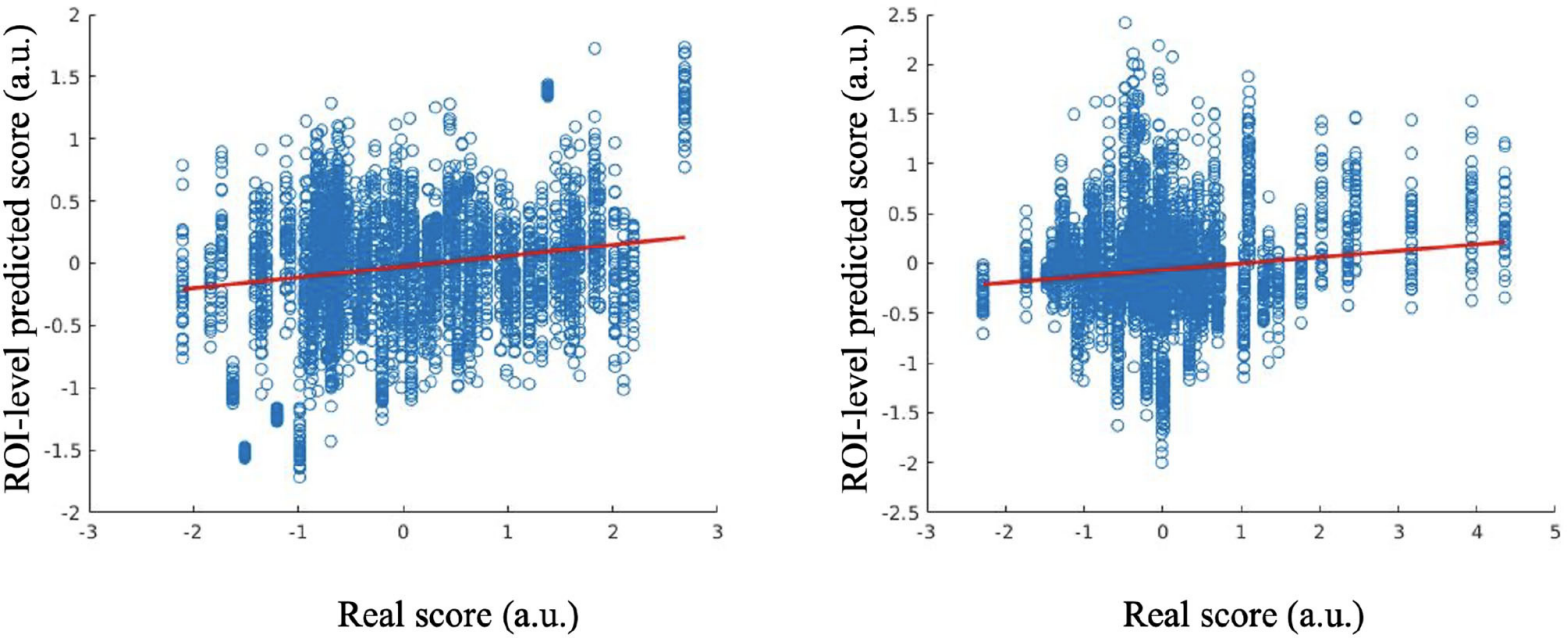
Estimated scores vs. real scores for Anxiety factor score at the ROI-level **(left)**; estimated scores vs. real scores for Suicidality factor score at the ROI-level **(right)**.

The predictive performance of the Anxiety factor score is shown in Figure 2, in which the red line indicates a robust regression-fitted line. At the ROI-level, the Pearson's correlation between the estimated and real scores was 0.2589 (*p* = 1.0152^*e*−61^), and the robust regression resulted in *t* = 11.5517 (*p* = 2.2200^*e*−30^). *P*-values were significant.

The results of estimated and real Suicidality factor scores are shown in Figure 2. At the ROI-level, the Pearson's correlation between the estimated and real scores is 0.1436 (*p* = 6.4760^*e*−20^), and the robust regression resulted in *t* = 9.3654 (*p* = 1.2392^*e*−20^). *P*-values were significant.

For the Appetite factor score, the Pearson's correlation between the estimated and real scores at the ROI-level evaluation was 0.0743 (*p* = 1.4763^*e*−06^), and the robust regression resulted in *t* = 5.2498 (*p* = 1.6029^*e*−07^).

For the Anhedonia factor, the Pearson's correlation between the estimated and real scores at the ROI-level was 0.0282 (*p* = 0.0384), and the robust regression resulted in *t* = 1.5794 (*p* = 0.1143).

For the Sleep Disturbance factor, Pearson's correlation between the estimated and real scores at the ROI level was −0.0836 (*p* = 7.3433^*e*−08^), and the robust regression resulted in *t* = −4.6128 (*p* = 4.0983^*e*−06^). The results for the Appetite, Anhedonia and Sleep Disturbance factor scores were not significant.

### 3.2. Contribution of ROI sets to factor scores

**Figure 3:**
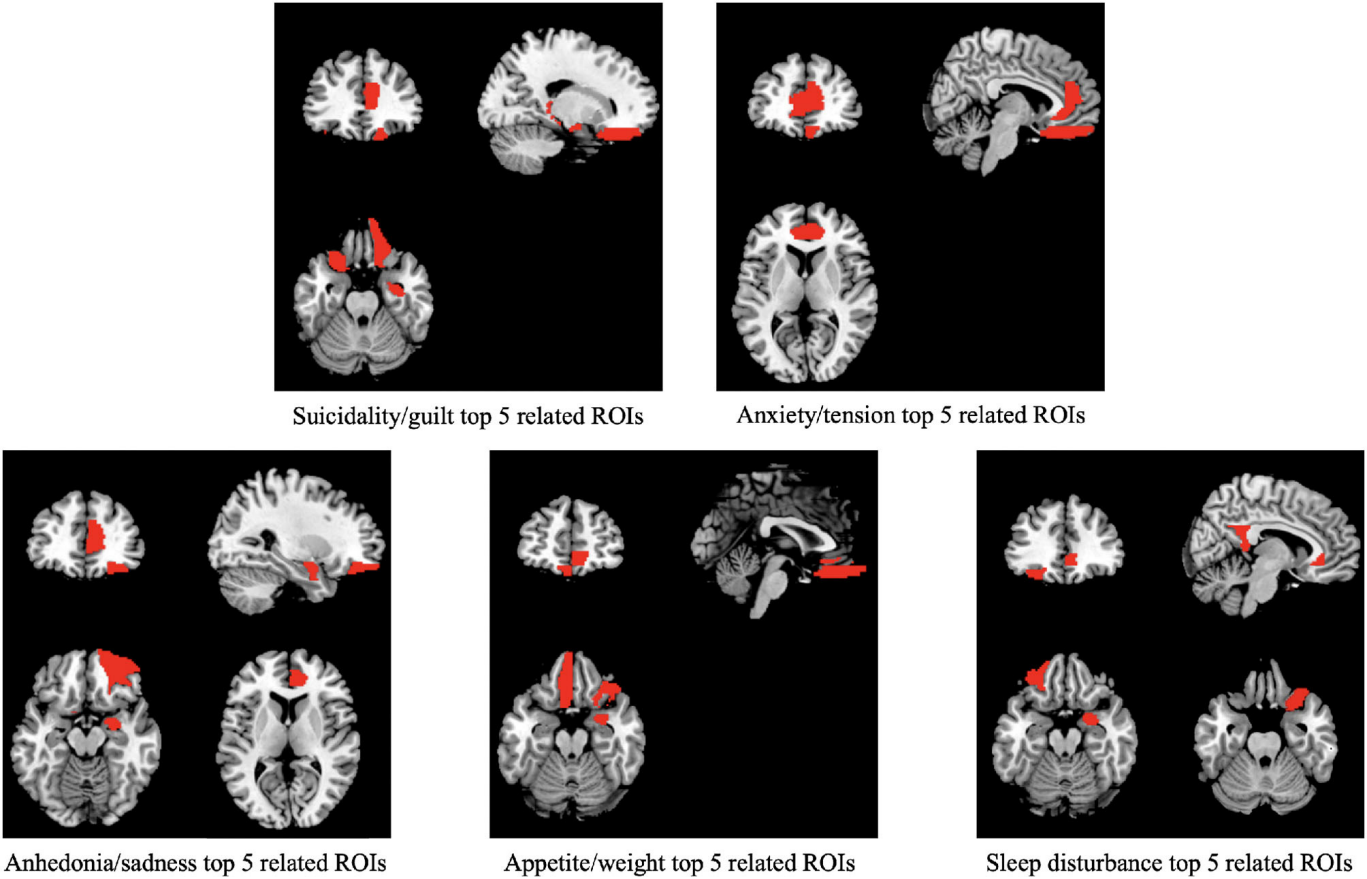
The top five important ROIs identified by our model in predicting five depression-related symptom factor scores.

We found that different ROIs tend to have different contributions to different prediction tasks. A lower median of the MSE reflects a more accurate estimate, which provides information about the potential importance of a given ROI set to a specific symptom factor score. Table 3 summarizes the top five most informative ROIs for each symptom factor, which shows that ACC and OFC are most informative to the prediction tasks compared to the other ROI sets. ACC is first ranked in four of the five symptom factor estimates, the OFC ROI set appears at least once in the top-five ranked ROI sets of all the factor score estimation models. Figure 3 presents the top five ROI sets of different estimation tasks overlaid on a standard brain template (Tzourio-mazoyer et al., 2002).

**Table 3:** Top five predictive ROIs by symptom factor score.

**Symptom factor**	**Rank 1**	**Rank 2**	**Rank 3**	**Rank 4**	**Rank 5**
Anxiety	ACCpre (L)	ACCpre (R)	REC (R)	ACCsub (R)	OFCpost (R)
Suicidality	ACCsup (R)	OFCmed (R)	HIP (R)	OFCpost (L)	IFGoperc (L)
Appetite	OFCmed (R)	AMYG (R)	Nacc (L)	Rect (L)	OFCpost (R)
Anhedonia	ACCpre (R)	Nacc (L)	AMYG (R)	OFCant (R)	ACCsub (R)
Sleep	ACCsub (R)	AMYG (R)	PCC (R)	OFCant (L)	IFGoperc (R)

Anatomical description for each abbreviation appears in Table 2.

## 4. Discussion

The goal of the current study was to use a deep learning approach to identify brain morphometric features in T1-weighted structural MRIs that are predictive of previously validated factor structure of depression symptom phenotypes. Among the five symptom phenotypes, our method yielded the best performance in estimating the Anxiety and Suicidality symptom factors with significant small-to-medium-sized correlations. Our findings suggest that in LLD, symptoms related to Anxiety and Suicidality may be more strongly related to brain morphological characteristics than symptoms related to anhedonia, appetite/weight loss, and sleep disturbance. We found features in the ACC and OFC had prominent predictive information for Anxiety and Suicidality factors. Our results suggest that deep-learning models such as ours can help identify deep embedded local morphological characteristics in raw structural MRI data to predict and characterize distinct symptom phenotypes of LLD, and can be accomplished with a modest sample size.

Considering this possible mechanism of results, we are hesitant to speculate beyond the data available to our study. A plausible explanation is that anxiety and suicidality are strongly linked to dysfunction in the emotional regulation of negative thoughts, which is largely cortically mediated, whereas the other symptom factors may have additional white matter and non-brain physiological contributors. A key mechanistic study by Etkin et al. (2010) used task-based fMRI to demonstrate that individuals with generalized anxiety disorder were unable to engage the pregenual ACC in ways that could help downregulate emotional conflict. Andreescu et al. (2015) reported results consistent with an emotional dysregulation explanation, including the anterior ACC in studies of anxious older adults in an antidepressant treatment trial. As with anxious symptoms, the role of dysfunctional emotional regulation in suicidality and LLD has been well-reviewed (Kiosses et al., 2014), with the cognitive control aspect of emotional regulation having greater impact in suicidality and LLD (Szanto et al., 2020), and which is more strongly localized to the default-mode network (Shao et al., 2021). Future work on phenotyping LLD symptoms using deep learning models should include multi-modal MRI and physiologic variables to more fully explore mechanistic hypotheses.

Our findings are consistent with the body of research showing that the ACC and OFC are core regions related to the expression of depression symptoms in LLD, and the predictive value was the strongest in the phenotype of anxious depression. A clinical phenotype of anxious depression has been estimated to compose 40–50% of individuals with LLD (Jeste et al., 2006). Anxious depression in LLD has been associated with greater severity of depression symptoms, more frequent suicidal ideation, and both attenuated and delayed responses to pharmacologic treatment (Andreescu et al., 2007). Anxiety symptoms in LLD have been found to have functional MRI correlates involving reduced connectivity between the anterior ACC regions and PFC (Gerlach et al., 2021), which is broadly consistent with the sMRI findings of our predictive model.

Our model did predict the Suicidality symptom phenotype with a small effect size. Although the predictive model identified features within the right dorsal ACC to be most informative, suicide risk in LLD is more prominently associated with the structural changes in prefrontal regions more than the ACC (Shao et al., 2021). We do note that features within the prefrontal cortex were also among the most predictive regions in our model.

Our models were not significantly predictive for symptom factors related to Anhedonia, Appetite/Weight Loss, or Sleep Disturbance. In the case of anhedonia symptoms in particular, this may reflect the fact that our model was based on T1 MRI scans and did not include white matter features. Anhedonia symptoms, such as lack of initiative, low energy, and psychomotor and cognitive slowing, are widely associated with white matter pathology in LLD (Aizenstein et al., 2016). White matter features would be expected to be important for the prediction of a vascular subtypes of LLD (Taylor et al., 2013). The addition of white matter features to machine learning models could improve prediction of anhedonia in LLD.

Identifying sMRI correlates of appetite and weight loss symptoms in LLD May be complicated by the fact that appetite and weight loss are signs of unhealthy aging in general (Gaddey and Holder, 2021), not just LLD, and the physiological mechanisms are complex (Lipsitz, 2004). Thus, heterogeneity of the symptom construct and overlap with aging pathologies may explain the lack of a significant predictive model. Other clinical markers, such as biomarkers of nutritional and metabolic change, could improve prediction of appetite loss in LDD.

Sleep disturbance, as characterized by insomnia, is a common symptom of depression in LLD; however, insomnia is also a symptom of many comorbid medical conditions present among older adults (Katz and McHorney, 1998). In addition, normal human aging is associated with changes in sleep architecture predisposing to reductions in sleep quality and quantity (Mander et al., 2017). Based on the heterogeneity of factors contributing to sleep loss in older adults, inclusion of white matter information from MRI and inclusion of clinical information on medical comorbidities like sleep apnea may improve deep learning prediction of sleep disturbance symptoms in LLD.

### 4.1. Clinical implications

The clinical implications of our study are most applicable to the estimated 40–50% of individuals with elevated anxiety as a comorbid feature of their LLD, which may be a phenotype that includes elevated suicide risk (Lenze et al., 2000). Older adults with anxious depression may be less responsive to antidepressant pharmacology (Andreescu et al., 2007), and therefore may need alternative or adjunctive approaches targeting the ACC and OFC, either directly or via functionally connected brain regions. Research suggests that function of the ACC, particularly the subgenual region, may be predictive of positive response to behavioral therapy in LLD (Solomonov et al., 2020). Another potential alternative or adjunctive treatment approach in LLD is transcranial magnetic stimulation, in which studies have demonstrated treatment success with major depressive disorder by targeting the ACC via its connectivity to the DLPFC (Weigand et al., 2017); however, further research is needed to test generalizability to LLD. With further refinement to include additional MRI and clinical information, deep learning models such as ours can be useful to defining symptom phenotypes in LLD to improve precision treatment selection.

### 4.2. Limitations and future work

The strengths of our study include a well-defined LLD sample combined with a novel deep learning approach that can be adapted to modest sample sizes. One limitation, however, was that we employed only the T1-weighted structural MRI data, without including other imaging modalities, such as T2-weighted, FLAIR, and functional resting state). Previous studies have shown that multimodal imaging data could contain complementary information for depression analysis (Gray et al., 2020), as well as integration of clinical data with multimodal imaging (Patel et al., 2015). Another limitation is that we focused on a set of a *priori* ROI regions informed by the research literature on LLD; however, it did not include some potentially informative regions (e.g., thalamus, middle temporal gyrus, fusiform gyrus, cerebellum). Future work with deep-learning models should integrate whole brain multiple imaging modalities and clinical data when there is adequate sample size to do so.

## 5. Conclusion

The novel findings of this study suggest that the proposed deep learning model has potential to predict depression symptom phenotypes in LLD based on T1-weighted structural MRI data with modest sample size requirements, but most effectively for an anxious-depression phenotype in LLD with a prominent predictive contribution from features in the ACC. Based on the promising findings of our deep-learning model in a small sample tot T1 MRI data, future research should replicate our approach with a larger sample size that facilitates modeling of multi-modal MRI and clinical markers.

## Data availability statement

The original contributions presented in the study are included in the article/supplementary material, further inquiries can be directed to the corresponding authors.

## Ethics statement

Ethical review and approval was not required for the study on human participants in accordance with the local legislation and institutional requirements. Written informed consent for participation was not required for this study in accordance with the national legislation and the institutional requirements.

## Author contributions

BC: designed and executed the analysis and co-wrote the manuscript. EY: data collection and analysis. LW: data analysis, interpretation, and critical revision of the manuscript. ZM: statistical analysis. HZ: designed the analysis, data analysis and interpretation, contribution of analysis tools, and critical revision of the manuscript. ML: designed the analysis, data analysis and interpretation, and critical revision of the manuscript. DS: conception of work, data analysis and interpretation, and critical revision of the manuscript. GP: conception of work, data analysis and interpretation, and co-wrote the manuscript. All authors contributed to the article and approved the submitted version.

## References

[B1] Aizenstein H. J. Baskys A. Boldrini M. Butters M. A. Diniz B. S. Jaiswal M. K.. (2016). Vascular depression consensus report97a critical update. BMC Med. 14:5. 10.1186/s12916-016-0720-5 27806704 PMC5093970

[B2] Andreescu C. Lenze E. J. Dew M. A. Begley A. E. Mulsant B. H. Dombrovski A. Y.. (2007). Effect of comorbid anxiety on treatment response and relapse risk in late-life depression: controlled study. Br. J. Psychiatry 190, 344–349. 10.1192/bjp.bp.106.027169 17401042

[B3] Andreescu C. Sheu L. K. Tudorascu D. Gross J. J. Walker S. Banihashemi L.. (2015). Emotion reactivity and regulation in late-life generalized anxiety disorder: functional connectivity at baseline and post-treatment. Am. J. Geriatr. Psychiatry 23, 200–214. 10.1016/j.jagp.2014.05.003 24996397 PMC4234701

[B4] Etkin A. Prater K. E. Hoeft F. Menon V. Schatzberg A. F. (2010). Failure of anterior cingulate activation and connectivity with the amygdala during implicit regulation of emotional processing in generalized anxiety disorder. Am. J. Psychiatry 167, 545–554. 10.1176/appi.ajp.2009.09070931 20123913 PMC4367202

[B5] Gaddey H. L. Holder K. K. (2021). Unintentional weight loss in older adults. Am. Fam. Phys. 104, 34–40. 34264616

[B6] Gao S. Calhoun V. D. Sui J. (2018). Machine learning in major depression: from classification to treatment outcome prediction. CNS Neurosci. Therap. 24, 1037–1052. 10.1111/cns.13048 30136381 PMC6324186

[B7] Gerlach A. R. Karim H. T. Kazan J. Aizenstein H. J. Krafty R. T. Andreescu C. (2021). Networks of worry–towards a connectivity-based signature of late-life worry using higher criticism. Transl. Psychiatry 11:550. 10.1038/s41398-021-01648-5 34711810 PMC8553743

[B8] Gray J. P. Müller V. I. Eickhoff S. B. Fox P. T. (2020). Multimodal abnormalities of brain structure and function in major depressive disorder: a meta-analysis of neuroimaging studies. Am. J. Psychiatry 2020:appiajp201919050560. 10.1176/appi.ajp.2019.19050560 32098488 PMC7294300

[B9] Gunning F. M. Oberlin L. E. Schier M. Victoria L. W. (2021). Brain-based mechanisms of late-life depression: implications for novel interventions. Semin. Cell Dev. Biol. 116, 169–179. 10.1016/j.semcdb.2021.05.002 33992530 PMC8548387

[B10] Hamilton M. (2000). “Hamilton rating scale for depression (Ham-d),” in Handbook of Psychiatric Measures, ed A. J. Rush (Washington, DC: American Psychiatric Association), 526–528.

[B11] He K. Zhang X. Ren S. Sun J. (2015). “Deep residual learning for image recognition,” in 2016 IEEE Conference on Computer Vision and Pattern Recognition (CVPR) (Las Vegas, NV), 770–778. 10.1109/CVPR.2016.90

[B12] Heo M. Murphy C. F. Meyers B. S. (2007). Relationship between the Hamilton depression rating scale and the Montgomery-Asberg depression rating scale in depressed elderly: a meta-analysis. Am. J. Geriatr. Psychiatry 15, 899–905. 10.1097/JGP.0b013e318098614e 17911366

[B13] Hybels C. F. Blazer D. G. Landerman L. R. Steffens D. C. (2011). Heterogeneity in symptom profiles among older adults diagnosed with major depression. Int. Psychogeriatr. 23, 906–922. 10.1017/S1041610210002346 21241529 PMC3139722

[B14] Ioffe S. Szegedy C. (2015). Batch normalization: accelerating deep network training by reducing internal covariate shift. ArXiv: abs/1502.03167. 10.48550/arXiv.1502.0316735496726

[B15] Jellinger K. A. (2022). Pathomechanisms of vascular depression in older adults. Int. J. Mol. Sci. 23:308. 10.3390/ijms23010308 35008732 PMC8745290

[B16] Jeste N. D. Hays J. C. Steffens D. C. (2006). Clinical correlates of anxious depression among elderly patients with depression. J. Affect. Disord. 90, 37–41. 10.1016/j.jad.2005.10.007 16325261

[B17] Joseph C. Wang L. Wu R. Manning K. J. Steffens D. C. (2021). Structural brain changes and neuroticism in late-life depression: a neural basis for depression subtypes. Int. Psychogeriatr. 33, 515–520. 10.1017/S1041610221000284 33762034 PMC8169547

[B18] Katz D. A. McHorney C. A. (1998). Clinical correlates of insomnia in patients with chronic illness. Arch. Intern. Med. 158, 1099–1107. 10.1001/archinte.158.10.1099 9605781

[B19] Kiosses D. N. Szanto K. Alexopoulos G. S. (2014). Suicide in older adults: the role of emotions and cognition. Curr. Psychiatry Rep. 16, 1–8. 10.1007/s11920-014-0495-3 25226883 PMC4225125

[B20] Lenze E. J. Mulsant B. H. Shear M. K. Schulberg H. C. Dew M. A. Begley A. E.. (2000). Comorbid anxiety disorders in depressed elderly patients. Am. J. Psychiatry 157, 722–728. 10.1176/appi.ajp.157.5.722 10784464

[B21] Lin E. Kuo P.-H. Liu Y.-L. Yu Y. W.-Y. Yang A. C. Tsai S.-J. (2018). A deep learning approach for predicting antidepressant response in major depression using clinical and genetic biomarkers. Front. Psychiatry 9:290. 10.3389/fpsyt.2018.00290 30034349 PMC6043864

[B22] Lipsitz L. A. (2004). Physiological complexity, aging, and the path to frailty. Sci. Aging Knowl. Environ. 16:pe16. 10.1126/sageke.2004.16.pe16 15103055

[B23] Litjens G. J. S. Kooi T. Bejnordi B. E. Setio A. A. A. Ciompi F. Ghafoorian M.. (2017). A survey on deep learning in medical image analysis. Med. Image Anal. 42, 60–88. 10.1016/j.media.2017.07.005 28778026

[B24] Ly M. Karim H. T. Becker J. T. Lopez O. L. Anderson S. J. Aizenstein H. J.. (2021). Late-life depression and increased risk of dementia: a longitudinal cohort study. Transl. Psychiatry 11:147. 10.1038/s41398-021-01269-y 33654078 PMC7925518

[B25] Lyness J. M. Niculescu A. Tu X. Reynolds C. F. Caine E. D. (2006). The relationship of medical comorbidity and depression in older, primary care patients. Psychosomatics 47, 435–439. 10.1176/appi.psy.47.5.435 16959933

[B26] Mander B. A. Winer J. R. Walker M. P. (2017). Sleep and human aging. Neuron 94, 19–36. 10.1016/j.neuron.2017.02.004 28384471 PMC5810920

[B27] Montgomery S. Åsberg M. (1979). A new depression scale designed to be sensitive to change. Br. J. Psychiatry 134, 382–389. 10.1192/bjp.134.4.382 444788

[B28] Nair V. Hinton G. E. (2010). “Rectified linear units improve restricted Boltzmann machines,” in International Conference on Machine Learning (Haifa).

[B29] Panza F. Frisardi V. Capurso C. D'Introno A. Colacicco A. M. Imbimbo B. P.. (2010). Late-life depression, mild cognitive impairment, and dementia: possible continuum? Am. J. Geriatr. Psychiatry 18, 98–116. 10.1097/JGP.0b013e3181b0fa13 20104067

[B30] Patel M. J. Andreescu C. Price J. C. Edelman K. L. Reynolds C. F. Aizenstein H. J. (2015). Machine learning approaches for integrating clinical and imaging features in late–life depression classification and response prediction. Int. J. Geriatr. Psychiatry 30, 1056–1067. 10.1002/gps.4262 25689482 PMC4683603

[B31] Pimontel M. A. Solomonov N. Oberlin L. Kanellopoulos T. Bress J. N. Hoptman M. J.. (2021). Cortical thickness of the salience network and change in apathy following antidepressant treatment for late-life depression. Am. J. Geriatr. Psychiatry 29, 241–248. 10.1016/j.jagp.2020.06.007 32680763 PMC7738363

[B32] Potter G. G. Mcquoid D. R. Steffens D. C. (2015). Appetite loss and neurocognitive deficits in late–life depression. Int. J. Geriatr. Psychiatry 30, 647–654. 10.1002/gps.4196 25315155 PMC4691536

[B33] Rolls E. T. Huang C.-C. Lin C.-P. Feng J. Joliot M. (2020). Automated anatomical labelling atlas 3. NeuroImage 206. 10.1016/j.neuroimage.2019.116189 31521825

[B34] Rolls E. T. Joliot M. Tzourio-Mazoyer N. (2015). Implementation of a new parcellation of the orbitofrontal cortex in the automated anatomical labeling atlas. NeuroImage 122, 1–5. 10.1016/j.neuroimage.2015.07.075 26241684

[B35] Shao R. Gao M. Lin C. Huang C.-M. Liu H.-L. Toh C. H.. (2021). Multimodal neural evidence on the corticostriatal underpinning of suicidality in late-life depression. Biol. Psychiatry Cogn. Neurosci. Neuroimaging. 7, 905–915. 10.1016/j.bpsc.2021.11.011 34861420

[B36] Solomonov N. Victoria L. W. Dunlop K. Respino M. Hoptman M. J. Oberlin L. E.. (2020). Resting state functional connectivity and outcomes of psychotherapies for late-life depression. Am. J. Geriatr. Psychiatry. 28:859–868. 10.31234/osf.io/uncsz 32376080 PMC7369214

[B37] Steffens D. C. Welsh-Bohmer K. Burke J. R. Plassman B. L. Beyer J. L. Gersing K.. (2004). Methodology and preliminary results from the neurocognitive outcomes of depression in the elderly study. J. Geriatr. Psychiatry Neurol. 17, 202–211. 10.1177/0891988704269819 15533991

[B38] Szanto K. Galfalvy H. Kenneally L. Almasi R. Dombrovski A. Y. (2020). Predictors of serious suicidal behavior in late-life depression. Eur. Neuropsychopharmacol. 40, 85–98. 10.1016/j.euroneuro.2020.06.005 32778367 PMC7655527

[B39] Taylor W. D. Aizenstein H. J. Alexopoulos G. S. (2013). The vascular depression hypothesis: mechanisms linking vascular disease with depression. Mol. Psychiatry 18, 963–974. 10.1038/mp.2013.20 23439482 PMC3674224

[B40] Tzourio-mazoyer N. Landeau B. Papathanassiou D. Crivello F. Etard O. Delcroix N.. (2002). Automated anatomical labeling of activations in SPM using a macroscopic anatomical parcellation of the MNI MRI single-subject brain. NeuroImage 15, 273–289. 10.1006/nimg.2001.0978 11771995

[B41] Uyulan Ç. Ergüzel T. T. Unubol H. Çebi M. Sayar G. H. Asad M. N.. (2020). Major depressive disorder classification based on different convolutional neural network models: deep learning approach. Clin. EEG Neurosci. 52, 38–51. 10.1177/1550059420916634 32491928

[B42] Veltman E. M. Lamers F. Comijs H. C. de Waal M. W. M. Stek M. L. van der Mast R. C.. (2017). Depressive subtypes in an elderly cohort identified using latent class analysis. J. Affect. Disord. 218, 123–130. 10.1016/j.jad.2017.04.059 28472702

[B43] Wassink-Vossen S. Collard R. M. Wardenaar K. J. Verhaak P. F. Rhebergen D. Naarding P.. (2019). Trajectories and determinants of functional limitations in late-life depression: a 2-year prospective cohort study. Eur. Psychiatry 62, 90–96. 10.1016/j.eurpsy.2019.09.003 31550583

[B44] Weigand A. Horn A. Caballero R. Cooke D. Stern A. P. Taylor S. F.. (2017). Prospective validation that subgenual connectivity predicts antidepressant efficacy of transcranial magnetic stimulation sites. Biol. Psychiatry 84, 28–37. 10.1016/j.biopsych.2017.10.028 29274805 PMC6091227

[B45] Zhang L. Yu M. Wang L. Steffens D. C. Wu R. Potter G. G.. (2022). Understanding clinical progression of late-life depression to Alzheimer's disease over 5 years with structural MRI. Machine Learn. Med. Imaging 13583, 259–268. 10.1007/978-3-031-21014-3_27 36594904 PMC9805302

[B46] Zhukovsky P. Anderson J. A. E. Coughlan G. Mulsant B. H. Cipriani A. Voineskos A. N. (2021). Coordinate-based network mapping of brain structure in major depressive disorder in younger and older adults: a systematic review and meta-analysis. Am. J. Psychiatry 2021:appiajp202121010088. 10.1176/appi.ajp.2021.21010088 34645274

[B47] Zhuo C. Li G. Lin X. Jiang D. Xu Y. Jun Tian H.. (2019). The rise and fall of MRI studies in major depressive disorder. Transl. Psychiatry 9:335. 10.1038/s41398-019-0680-6 31819044 PMC6901449

